# GC/MS Evaluation and In Vitro Antioxidant Activity of Essential Oil and Solvent Extracts of an Endemic Plant Used as Folk Remedy in Turkey: *Phlomis bourgaei* Boiss.

**DOI:** 10.1155/2013/293080

**Published:** 2013-05-13

**Authors:** Cengiz Sarikurkcu, M. Sabih Ozer, Ahmet Cakir, Mustafa Eskici, Ebru Mete

**Affiliations:** ^1^Department of Gastronomy and Culinary Arts, Faculty of Tourism, University of Necmettin Erbakan, Meram, Konya 42100, Turkey; ^2^Department of Chemistry, Faculty of Science and Literature, Celal Bayar University, Muradiye, Manisa 45030, Turkey; ^3^Department of Chemistry, Faculty of Arts and Sciences, Kilis 7 Aralık University, Kilis 79000, Turkey; ^4^Department of Chemistry, Faculty of Science, Atatürk University, Erzurum 25240, Turkey

## Abstract

This study was outlined to examine the chemical composition of hydrodistilled essential oil and in vitro antioxidant potentials of the essential oil and different solvent extracts of endemic *Phlomis bourgaei* Boiss. used as folk remedy in Turkey. The chemical composition of the oil was analyzed by GC and GC-MS, and the predominant components in the oil were found to be **β**-caryophyllene (37.37%), *(Z)*-**β**-farnesene (15.88%), and germacrene D (10.97%). Antioxidant potentials of the solvent extracts and the oil were determined by four testing systems including **β**-carotene/linoleic acid, DPPH, reducing power, and chelating effect. In **β**-carotene/linoleic acid assay, all extracts showed the inhibition of more than 50% at all concentrations. In DPPH, chelating effect, and reducing power test systems, the water extract with 88.68%, 77.45%, and 1.857 (absorbance at 700 nm), respectively, exhibited more excellent activity potential than other extracts (hexane, ethyl acetate and methanol) and the essential oil at 1.0 mg/mL concentration. The amount of the total phenolics and flavonoids was the highest in this extract (139.50 ± 3.98 **μ**g gallic acid equivalents (GAEs)/mg extract and 22.71 ± 0.05 **μ**g quercetin equivalents (QEs)/mg extract).

## 1. Introduction

Lipid peroxidation is one of the major factors causing deterioration of foods during the storage and processing. Also, oxidized polyunsaturated fatty acids may induce aging and carcinogenesis [1]. Although there are some synthetic antioxidant compounds, such as butylated hydroxytoluene (BHT) and butylated hydroxyanisole (BHA), which are commonly used in processed foods, it has been reported that these compounds can have some side effects [2]. Therefore, many researchers have focused their attention on natural antioxidants. Reactive oxygen species (ROS) and reactive nitrogen species (RNS) are produced in cells by different means [3]. Exogenous sources of free radicals include tobacco smoke, ionizing radiation, certain pollutants, organic solvents, and pesticides [1]. ROS and RNS may also cause DNA damage that could lead to mutation. All aerobic organisms, including humans, have antioxidant defenses that protect against oxidative damage [3]. However, these natural antioxidant mechanisms can be inefficient, and hence dietary intake of antioxidant compounds becomes important. Herbal therapy has been widely used to treat a large variety of various ailments and symptoms, and there is a recently growing interest on the herbal therapy in the world. It has also been believed that medicinal plants are an important source of alternative chemical reagents with potential therapeutic effects [4, 5]. One of the potential uses of plant-derived compounds is to be used as antioxidant agents [3, 6, 7]. It is well known that antioxidants may be useful in protecting from cancer and other mutation-related diseases. The hypothesis is that the use of antioxidants that scavenge reactive oxygen species (ROS) provides biological resistance to free radicals, retards the process of aging, and decreases the risk of age-associated degenerative diseases, such as cancer, cardiovascular diseases, immune system decline, and brain dysfunction [5].

The genus *Phlomis* as perennial herbs of Lamiaceae family consists of more than 100 species distributed in Europe, Asia, and Africa. In Turkish flora, 52 taxa include 6 varieties, 12 natural hybrids, 34 endemic taxa which are distributed [8]. Many species of this genus have usage for medicinal and aromatic purposes. The aerial parts of some species including *Phlomis* and* Thymus *have distinctive tastes and are used for the herbal tea in traditional medicine as stimulants, tonics, carminative, appetizer, andiuretics [4]. It has been shown that *Phlomis* species have exhibited the various biolodgical properties such as in treatment of ulcer and hemorrhoids [7, 9, 10]. It was also reported that some *Phlomis* species have anti-inflammatory, antimicrobial [9], antiulcerogenic [10], immunosuppressive [11], and free radical scavenging [12] activities. Pytochemical investigations of *Phlomis* species were the subject to several studies, and, consequently, iridoid and phenyl propanoid glycosides were isolated [7, 13].

The *Phlomis* genus and their essential oils are used in the food and pharmaceutical industries. Essential oils isolated from *Phlomis* species are used as flavoring for foods and as fragrance in the perfume and cosmetic industry. Therefore, there were numerous reports on the chemical analyses of the essential oils of various *Phlomis* species distributed in different regions of the world [7, 8, 15]. On the other hand, as far as our literature survey could ascertain, there is only one report on the chemical composition of the essential oil of *P. bourgaei* [16]. Additionally, antioxidant activities of the solvent extracts and the essential oil of this plant have not previously been reported. Therefore, data presented here will be the first record on *P. bourgaei*.

The aim of this study is to determine the potential antioxidant and free radical scavenging activities, reducing and chelating effects of hexane, ethyl acetate, methanol and water extracts, and essential oil of *P. bourgaei* as well as the chemical composition of the oil. Additionally, total phenolic and flavonoid contents of the extracts have been determined.

## 2. Materials and Methods

### 2.1. Plant Material

*Phlomis bourgaei *Boiss. was collected in 2008 from Mugla University campus, Mugla, Turkey, at its flowering season. Taxonomic identification of the plant material was confirmed by the senior taxonomists Dr. Omer Varol and Dr. Olcay Ceylan, in Department of Biology, Mugla University. The voucher specimen has been deposited at the Herbarium of the Department of Biology, Mugla University, Mugla,Turkey (Voucher number OC 0652).

### 2.2. Preparation of the Extracts

For extraction, four different solvents (hexane, ethyl acetate, methanol, and water) were used to fractionate the soluble compounds from the *P. bourgaei *in ascending polarity.

The air-dried sample (20 g) of the aerial part of the plant was extracted by using a Sohxlet extractor for 5 h with 250 mL of hexane, ethyl acetate, and methanol under reflux conditions. The residue was then extracted by boiling water (300 mL). Hexane, ethyl acetate, and methanol in the extracts were removed with a rotary evaporator to obtain the extracts in the yield of 1.44%, 0.75%, and 7.17% (w/w), respectively. The water extract was dried in a freeze drier to obtain an extract in the yield of 11.37% (w/w).

### 2.3. Isolation of the Essential Oil

The air-dried and ground plant material (500 g) was submitted for 5 h to water distillation using a British-type Clevenger apparatus (ILDAM Ltd., Ankara, Turkey) (yield 0.11%). The obtained essential oil was dried over anhydrous sodium sulphate and after filtration, stored at +4°C until tested and analyzed.

### 2.4. Gas Chromatography (GC) Analysis

The analysis of the essential oil was performed using a Thermo Finnigan Trace GC/A1300, (E.I), equipped with a SGE/BPX5 MS capillary column (30 m × 0.25 mm i.d., 0.25 *μ*m). Helium was the carrier gas, at a flow rate of 1 mL/min. Injector temperature was set at 220°C. The programme used was 50–150°C at a rate of 3°C/min, held isothermal for 10 min, and finally raised to 250°C at 10°C/min. Diluted samples (1/100, v/v, in methylene chloride) of 1.0 *μ*L were injected manually and in the splitless mode. Quantitative data of the oil was obtained from flame ionization detector (FID) area percentage data.

### 2.5. Gas Chromatography/Mass Spectrometry (GC/MS) Analysis

The analysis of the essential oil was performed with a Thermo Finnigan Trace GC/Trace DSQ/A1300, (E.I Quadrapole) equipped with a SGE-BPX5 MS fused silica capillary column (30 m × 0.25 mm i.d., film thickness 0.25 *μ*m). For GC-MS detection, an electron ionization system with ionization energy of 70 eV was used. Carrier gas was helium at a flow rate of 1 mL/min. Injector and MS transfer line temperatures were set at 220°C and 290°C, respectively. The oven temperature was programmed from 50°C to 150°C at 3°C/min, then held isothermal for 10 min, and raised to 250°C at 10°C/min. Diluted samples (1/100, v/v, in methylene chloride) of 1.0 *μ*L were injected manually in the splitless mode.

The identification of individual compounds was based on comparison of their relative retention times with those of authentic samples on SGE-BPX5 capillary column, and by matching their mass spectra of peaks with those obtained from authentic samples and/or the Wiley 7N and TRLIB libraries spectra and published data [14].

### 2.6. Antioxidant Activity

#### 2.6.1. Total Antioxidant Activity by *β*-Carotene-Linoleic Acid Method

In this assay antioxidant capacity is determined by measuring the inhibition of the volatile organic compounds and the conjugated diene hydroperoxides arising from linoleic acid oxidation [17]. A stock solution of *β*-carotene-linoleic acid mixture was prepared as follows: 0.5 mg *β*-carotene was dissolved in 1 mL of chloroform (HPLC grade). Twenty-five microliters of linoleic acid and 200 mg Tween 40 were added. Chloroform was completely evaporated using a vacuum evaporator. Then 100 mL of oxygenated distilled water was added with vigorous shaking; 2.5 mL of this reaction mixture was dispersed to test tubes, 0.5 mL of various concentrations (0.4–2.0 mg/mL) of the extracts in methanol and water was added, and the emulsion system was incubated for up to 2 h at 50°C. The same procedure was repeated with the positive control BHT, BHA, and a blank. After this incubation period, absorbance of the mixtures was measured at 490 nm. Measurement of absorbance was continued until the color of *β*-carotene disappeared. The bleaching rate (*R*) of *β*-carotene was calculated according to

(1) R=[ln⁡(a/b)t],

where ln = natural log, *a *= absorbance at time 0, and *b *= absorbance at time *t* (30, 60, 90, and 120 min). The antioxidant activity (*AA*) was calculated in terms of percent inhibition relative to the control using

(2) AA=[(RControl−RSample)RControl]×100.

Antioxidant activities of the extracts were compared with those of BHT and BHA at 0.4 mg/mL and blank consisting of only 0.5 mL methanol and water.

#### 2.6.2. Scavenging Capacity on 1,1-Diphenyl-2-picrylhydrazyl (DPPH) Radical

The hydrogen atoms or electrons donation ability of the corresponding extracts and some pure compounds was measured from the bleaching of purple-colored methanol solution of DPPH. The effect of the extracts on DPPH radical was estimated according to Hatano et al. [18]. One milliliter of various concentrations (0.2–1.0 mg/mL) of the extracts in methanol and water was added to 1 mL of DPPH radical solution in methanol (final concentration of DPPH was 0.2 mM). After a 30 min incubation period at room temperature the absorbance was read against a blank at 517 nm. Inhibition of free-radical DPPH in percent (*I*%) was calculated according to (3):

(3) I%=(AControl−ASample)AControl×100,

where *A*_Control_ is the absorbance of the control reaction (containing all reagents except the test compound) and *A*_Sample_ is the absorbance of the compound tested. BHT and BHA were used as a control.

#### 2.6.3. Reducing Power

The reducing power was determined according to the method of Oyaizu [19]. Each of the extracts (0.2–1.0 mg/mL) in methanol and water (2.5 mL) was mixed with 2.5 mL of 200 mM sodium phosphate buffer (pH 6.6) and 2.5 mL of 1% potassium ferricyanide. Reaction mixture was incubated at 50°C for 20 min, and then 2.5 mL of 10% trichloroacetic acid was added. The mixture was centrifuged at 3000 rpm (MSE Mistral 2000, London, UK) for 10 min. The upper layer (2.5 mL) was mixed with 2.5 mL of deionized water and 0.5 mL of 0.1% ferric chloride, and the absorbance was measured at 700 nm against a blank. BHT and BHA were used as a control.

#### 2.6.4. Chelating Effects on Ferrous Ions

The chelating effect was determined according to the method of Dinis et al. [20]. Briefly, 2 mL of the various concentrations (0.25–1.00 mg/mL) of the extracts in methanol and water was added in 2 mM FeCl_2_ solution (0.05 mL). The reaction was initiated by the addition of 5 mM ferrozine (0.2 mL). Then, the mixture was shaken vigorously and left at room temperature for 10 min. Absorbance of the solution was measured spectrophotometrically at 562 nm. The inhibition percentage of ferrozine-Fe^2+^ complex formation was calculated according to (4):

(4) metal  chelating  effect  (%)=[(AControl−ASample)AControl]×100,

where *A*_Control_ is the absorbance of control and *A*_Sample_ is the absorbance of the compound tested. EDTA was used as the control agent.

#### 2.6.5. Assay for Total Phenolics

Phenolic contents of the extracts were determined by employing the methods given in the literature [21]. One milliliter of the extract solution containing 2 mg extract was added to a volumetric flask. Then, 45 mL distilled water and 1 mL Folin-Ciocalteu reagent were added and flask was shaken vigorously. After 3 min, a 3 mL solution of Na_2_CO_3_ (2%) was added and the mixture was allowed to stand for 2 h with intermittent shaking. Absorbance was measured at 760 nm. The concentrations of phenolic compounds were calculated according to the following equation (5) obtained from the standard gallic acid graph:

(5) Absorbance=0.0162  gallic  acid (μg)−0.0053 (R2:0.9993).

#### 2.6.6. Assay for Total Flavonoids

Flavonoid contents of the extracts were determined using the Dowd method as adapted by Arvouet-Grand et al. [22]. Briefly, 1 mL of 2% aluminium trichloride (AlCl_3_) in methanol was mixed with the same volume of the solvent extracts. Absorbance values were measured at 415 nm after a 10 min resting against a blank sample containing 1 mL of extract solution with 1 mL methanol (without AlCl_3_). Concentrations of flavonoid compounds were calculated according to the following equation (6) obtained from the standard quercetin graph:

(6) absorbance=0.0274  quercetin (μg)+0.0012 (R2:0.9991).

## 3. Results and Discussion

### 3.1. Chemical Composition of the Essential Oil

The hydrodistillation of the flowering aerial parts of *P. bourgaei* gave yellowish oil with a yield of 0.11% (w/w). The components identified in the oil, their retention indices, and relative percentages are listed in Table 1. The GC and GC-MS analyses of *P. bourgaei* oil resulted in detection of 13 components representing 95.75% of the total oil (Table 1). As shown in Table 1, the oil was characterized by relatively high content of sesquiterpene hydrocarbons representing 79.20% of the oil. *β*-Caryophyllene (37.37%), (*Z*)-*β*-farnesene (15.88%), germacrene D (10.97%), **α**-cubebene (7.43%), and *α*-copaene (2.19%) are the major sesquiterpene constituents of the oil. Furthermore, oxygenated sesquiterpenes were not detected in the essential oil of Turkish *P. bourgaei* in the present study. Limonene (8.97%) and **α**-pinene (5.65%) were found to be predominant monoterpenes in *P. bourgaei *oil (Table 1).

As far as our literature survey could ascertain, one report on chemical composition of *P. bourgaei* essential oil has been previously reported [16]. In this report, the essential oil of Turkish *P. bourgaei* was characterized by the high content of sesquiterpenes, and germacrene D (11.3%) and *β*-caryophyllene (11.2%) are the major components of this oil. As can be seen from Table 1, these results are in agreement with our findings. It has also been documented that the essential oils of the genus *Phlomis* are rich in sesquiterpenes with different chemical composition [8, 23–25]. According to these reports, in general, the essential oils of various *Phlomis* species have been characterized by high content of germacrene D, **β**-caryophyllene, (*Z*)- and (*E*)-**β**-farnesene, **β**-selinene, bicyclogermacrene, **α**-pinene, and limonene [8, 23–25]. Nevertheless, in the current study, **β**-selinene and bicyclogermacrene, which were found to be major components in the essential oil of some *Phlomis* species, were not detected in the oil of *P. bourgaei*. On the other hand, some chemotypes exhibited different chemical composition. Zhang and Wang [26] documented that *P. umbrosa* and *P. megalantha* contain hexadecanoic acid (52.1% and 46.0%, resp.), 9,12,15-octadecatrien-1-ol (24.8% and 22.6%, resp.), and *trans*-phytol (5.7% and 6.2%, resp.) as predominant components. This report also showed that *trans*-phytol (50.8%), 9,12,15-octadecadienoic acid methyl ester (11.0%), hexahydrofarnesyl acetone (8.5%), and hexadecanoic acid (7.1%) are the major constituents of Chinese *P. szechuanensis* essential oil [26]. According to another study, *P. fruticosa* growing in Serbia showed a different chemotype which is rich in **α**-pinene (56.6%), 1,8-cineole (10.48%), **α**-thujene (2.30%), limonene (2.2%), and **β**-caryophyllene (2.0%) [15].

### 3.2. Antioxidant Properties

Antioxidant potentials of the essential oil and the extracts of *P. bourgaei* were determined by four different test systems, namely, **β**-carotene-linoleic acid, DPPH, reducing power, and chelating effect.

The antioxidant activities of the plant extracts and the oil were evaluated by the spectrophotometric **β**-carotene bleaching test. In this method, **β**-carotene undergoes rapid discoloration in the absence of an antioxidant. **β**-Carotene bleaching method is based on the loss of the yellow color of **β**-carotene, which is monitored spectrophotometrically, due to its reaction with free radicals formed by linoleic acid oxidation in the emulsion system. The rate of **β**-carotene bleaching slowed down in the presence of antioxidants [27]. The inhibition values (%) of linoleic acid oxidation of the extracts and the oil at 0.4, 1.0 and 2.0 mg/mL concentrations are shown in Table 2. The results showed that antioxidant activity of both the extracts and the oil increased at dose-dependent manner. Among the extracts, the hexane and water extracts have the highest inhibition effect on the linoleic acid oxidation (Table 2). At the lowest concentration (0.4 mg/mL), the hexane extract had 76.96% antioxidant activity. However, the lowest antioxidant activity was obtained in the essential oil at all concentrations tested.

The effect of antioxidant on DPPH radical scavenging was thought to be due to their hydrogen-donating ability or radical scavenging activity. When a solution containing DPPH radicals is mixed with that of an antioxidant that may donate a hydrogen atom, afterward this gives rise to the reduced form of DPPH, which is nonradical with discoloration of this violet color [27]. Thus, the degree of discoloration indicates the free radical scavenging potentials of the samples.  Table 3 demonstrates the DPPH radical scavenging activity, expressed in percentage, of the various organic extracts and the essential oil of *P. bourgaei*. As can be seen from Table 3, DPPH radical scavenging effects of the samples tested exhibited a dose-dependent increase except for the water extract. The essential oil and the hexane extract with inhibition of less than 10% at all concentrations showed weaker DPPH radical scavenging activity than other extracts and standards. Nevertheless, different results were obtained for DPPH radical scavenging activities of the extracts compared to those obtained for antioxidant activity (Table 2). It is also interesting to note that the hexane extract, which has strong antioxidant activity, showed a weak DPPH radical scavenging activity. Low DPPH radical scavenging activity of the hexane extract may be regard, with its solubility in emulsion system. Water was found to be the best solvent for extracting the DPPH radical scavenging components from the plant samples compared to hexane, ethyl acetate, and also methanol. It scavenged the DPPH radicals with ratio of 86.90–90.42% (Table 3). Hence, it can be suggested that polar compounds present in the plant sample are mainly responsible for its free radical scavenging activity. It seems that the results from the DPPH radical scavenging activities of the plant samples do not always correlate with their antioxidant activities.

Previous reports pointed out that the electron donation capacity reflects the reducing power of the samples in association with antioxidant activity. Inactivation of oxidants by reduction with antioxidants can be defined as a redox reaction in which one reaction species is reduced at the expense of the oxidation of the other. In the reducing power assays, increasing absorbance at 700 nm indicates an increase in reducing power.  Table 4 presents the reducing power of the essential oil and the extracts of *P. bourgaei*. It was found that the reducing power of the extracts and the oil increased with the increase of their concentrations. As can be seen from Tables 3 and 4, the similar results were also observed for the reducing power of the extracts and the oil as compared to their DPPH-scavenging activities. The present results showed that the water extract was superior to other extracts in terms of reducing power. The reducing powers of the water extract were 0.526, 0.929, and 1.857 at 0.2, 0.4, and 1.0 mg/mL, respectively.

Metal ions can initiate lipid peroxidation and start a chain reaction that leads to the deterioration of food [28]. The catalysis of metal ions also correlates with incidents of cancer and arthritis. Ferrous ions, the most effective prooxidants, are commonly found in food systems. Iron can stimulate lipid peroxidation by the Fenton reaction, and also accelerates peroxidation by decomposing lipid hydroperoxides into peroxyl and alkoxyl radicals that can themselves abstract hydrogen and perpetuate the chain reaction of lipid peroxidation [29].  Table 5 presents the chelating abilities of the extracts and the essential oil. They exhibited various chelating effects on ferrous ion in a dose-dependent manner except for ethyl acetate extract (Table 5). While water extract and the essential oil have the highest chelating effect, the ethyl acetate and methanol extracts showed lower chelating activity.

It is well known that plant phenolics and/or polyphenolics constitute one of the major groups of plant-derived compounds acting as primary antioxidants against reactive free radicals [6, 7, 12, 20]. Flavonoids, as one of the most diverse and widespread kinds of natural phenolics, possess various biological activities including radical scavenging and antioxidant activities [6, 7, 18]. Hence, total phenolics and flavonoids contents present in the extracts of *P. bourgaei* were determined in the current study, and the results are shown in Table 6. Polar solvents, methanol, and water were found to be the best solvents for extracting the phenolics and flavonoids from the plant sample. These results would clearly suggest that, in general, there is a correlation between antioxidant potential and total phenolic content of the extracts. On the other hand, the hexane extract showed potent inhibition effect on the oxidation of linoleic acid, whereas it contains relatively low amount of phenolics (Table 6). Therefore, the antioxidant potential of the hexane extract of the plant sample can be attributed to its nonphenolic components. Likewise, the ethyl acetate extract, containing relatively high amounts of phenolics, has relatively low antioxidant and free radical scavenging activities (Tables 2 and 3).

Consequently, reports have demonstrated that some* Phlomis* species contain phenolic compounds such as flavonoids, phenolic acids, phenylethyl alcohols, and tannins [5, 7, 13]. Thus, the antioxidant potential of *P. bourgaei* tested in the current study may be attributed to its phenolic compounds, particularly in the methanol and water extracts. Recently, similar results for antioxidant potentials of some *Phlomis* species growing in different regions of the world have been documented [5, 7, 12, 30].

## 4. Conclusion

This study can be considered as the first report on the antioxidant potentials of the essential oil and various organic solvents extracts of *Phlomis bourgaei* from Turkey. The current results showed that *P. bourgaei* has potent antioxidant potentials and chelating effects. It can be concluded that this plant sample may find new benefits as natural sources in the food and pharmaceutical industries. However, further studies are needed to clarify bioactive compounds. This will be the aim of our further studies.

## Figures and Tables

**Table 1:** Chemical composition of the essential oil of aerial parts of *Phlomis bourgaei*.

Number	RI^a^	RT	Components	(%)	Identification methods
1	938	10.02	*α*-Pinene	5.65	GC, MS, RI
2	1037	14.52	Limonene	8.97	GC, MS, RI
3	1100	18.14	Perillene	1.93	MS, RI
4	1340	29.28	*α*-Cubebene	7.43	GC, MS, RI
5	1373	30.61	*α*-Copaene	2.19	GC, MS, RI
6	1383	30.97	*β*-Bourbonene	tr	GC, MS, RI
7	1387	31.17	Isolongifolene	1.80	GC, MS, RI
8	1419	32.57	*β*-Caryophyllene	37.37	GC, MS, RI
9	1453	33.83	(*Z*)-*β*-Farnesene	15.88	GC, MS, RI
10	1460	34.12	*α*-Humulene	0.86	GC, MS, RI
11	1486	36.21	Germacrene D	10.97	GC, MS, RI
12	1494	35.61	*δ*-Selinene	1.51	MS, RI
13	1517	36.70	*δ*-Cadinene	1.19	GC, MS, RI

Grouped components					(%)
Monoterpene hydrocarbons					14.62
Oxygenated monoterpenes					1.93
Sesquiterpene hydrocarbons					79.20
Oxygenated sesquiterpenes					—
Others					—

Total					95.75

^a^Retention index (RI) relative to* n*-alkanes on SGE-BPX5 capillary column.

RT: retention time (minute); GC: coinjection with standards; MS: tentatively identified based on computer matching of the mass spectra of peaks with Wiley 7N and TRLIB libraries and published data [14]; RI: identification based on comparison of retention index with those of published data [14]; tr: traces (less than 0.1%).

**Table 2:** Antioxidant activity (%) of the essential oil and the extracts from *P. bourgaei* at different concentrations measured by *β*-carotene–linoleic acid method^a^.

Sample	Sample concentration (mg/mL)		
	0.4	1.0	2.0
Essential oil	55.46 ± 1.86	65.88 ± 3.53	70.62 ± 2.37
Hexane	76.96 ± 0.88	85.56 ± 1.07	90.31 ± 0.24
Ethyl acetate	58.83 ± 0.46	72.17 ± 5.86	75.56 ± 2.67
Methanol	69.28 ± 1.66	80.19 ± 0.73	82.62 ± 1.76
Water	67.42 ± 0.59	83.11 ± 8.99	93.55 ± 2.58
BHT	92.77 ± 0.19		
BHA	92.34 ± 0.13		

^a^Values expressed are means ± S.D. of three parallel measurements.

**Table 3:** Scavenging effect (%) on 1,1-diphenyl-2-picrylhydrazyl of the essential oil and the extracts from *P. bourgaei* at different concentrations^a^.

Sample	Sample concentration (mg/mL)		
	0.4	1.0	2.0
Essential oil	2.02 ± 0.90	4.45 ± 0.36	9.02 ± 0.45
Hexane	2.33 ± 0.22	3.44 ± 0.28	8.37 ± 0.53
Ethyl acetate	20.68 ± 0.53	36.68 ± 0.01	73.33 ± 2.13
Methanol	18.24 ± 0.11	35.08 ± 0.03	82.21 ± 1.37
Water	90.42 ± 0.28	88.68 ± 0.06	86.90 ± 0.45
BHT	93.85 ± 1.59		
BHA	96.12 ± 1.17		

^a^Values expressed are means ± S.D. of three parallel measurements.

**Table 4:** Reducing power (absorbance at 700 nm) of the essential oil and the extracts from *P. bourgaei* at different concentrations^a^.

Sample	Sample concentration (mg/mL)		
	0.2	0.4	1.0
Essential oil	0.099 ± 0.006	0.199 ± 0.008	0.446 ± 0.004
Hexane	0.051 ± 0.003	0.078 ± 0.001	0.166 ± 0.016
Ethyl acetate	0.248 ± 0.009	0.478 ± 0.012	0.987 ± 0.021
Methanol	0.184 ± 0.009	0.347 ± 0.033	0.799 ± 0.021
Water	0.526 ± 0.030	0.929 ± 0.029	1.857 ± 0.020
BHT	2.938 ± 0.387	—	—
BHA	2.440 ± 0.028	—	—

^a^Values expressed are means ± S.D. of three parallel measurements.

**Table 5:** Chelating effect (%) of the essential oil and the extracts from *P. bourgaei* at different concentrations^a^.

Samples	Sample concentration (mg/mL)		
	0.25	0.50	1.00
Essential oil	43.96 ± 3.82	52.26 ± 0.57	63.84 ± 0.24
Hexane	22.01 ± 0.19	30.96 ± 2.91	38.42 ± 0.01
Ethyl acetate	7.19 ± 4.25	9.05 ± 0.57	9.18 ± 0.67
Methanol	2.40 ± 0.14	7.93 ± 1.00	48.04 ± 0.53
Water	59.18 ± 1.00	67.89 ± 0.33	77.45 ± 0.95
EDTA	99.36 ± 0.05	—	—

^a^Values expressed are means ± S.D. of three parallel measurements.

**Table 6:** Total phenolics and flavonoids content of the extracts from *P. bourgaei* ^a^.

Sample	Phenolic content (*µ*g GAEs/mg extract)^b^	Flavonoid content (*µ*g QEs/mg extract)^c^
Hexane	30.69 ± 1.12	1.69 ± 0.05
Ethyl acetate	94.55 ± 5.38	3.82 ± 0.06
Methanol	56.61 ± 3.65	19.91 ± 0.45
Water	139.50 ± 3.98	22.71 ± 0.05

^a^Values expressed are means ± S.D. of three parallel measurements.

^b^GAEs: gallic acid equivalents.

^c^QEs: quercetin equivalents.

## Abbreviations

BHA: Butylated hydroxyanisole
BHT: Butylated hydroxytoluene
DPPH: 1,1-Diphenyl-2-picrylhydrazyl
GAEs: Gallic acid equivalents
GC: Gas chromatography
GC/MS: Gas chromatography—mass spectrometry
QEs: Quercetin equivalents.

## References

[1] Halliwell B, Gutteridge JM (1989). *Free Radicals in Biology and Medicine*.

[2] Ito N, Fukushima S, Hassegawa A, Shibata M, Ogiso T (1983). Carcinogenecity of butylated hydroxyanisole in F344 ratsş. *Journal of the National Cancer Institute*.

[3] Davies KJA (2000). Oxidative stress, antioxidant defenses, and damage removal, repair, and replacement systems. *IUBMB Life*.

[4] Baytop T (1999). *Therapy with Medicinal Plants in Turkey; Today and in Future*.

[5] Dellai A, Mansour HB, Limem I (2009). Screening of antimutagenicity via antioxidant activity in different extracts from the flowers of *Phlomis crinita* Cav. ssp *Mauritanica munby* from the center of Tunisia. *Drug and Chemical Toxicology*.

[6] Çakir A, Mavi A, Kazaz C, Yildirim A, Küfrevioğlu OI (2006). Antioxidant activities of the extracts and components of *Teucrium orientale* L. var. *orientale*. *Turkish Journal of Chemistry*.

[7] Zhang Y, Wang ZZ (2009). Phenolic composition and antioxidant activities of two *Phlomis* species: a correlation study. *Comptes Rendus Biologies*.

[8] Demirci F, Guven K, Demirci B, Dadandi MY, Baser KHC (2008). Antibacterial activity of two *Phlomis* essential oils against food pathogens. *Food Control*.

[9] Couladis M, Tanimanidis A, Tzakou O, Chinou IB, Harvala C (2000). Essential oil of *Phlomis lanata* growing in Greece: chemical composition and antimicrobial activity. *Planta Medica*.

[10] Gürbüz I, Üstün O, Yesilada E, Sezik E, Kutsal O (2003). Anti-ulcerogenic activity of some plants used as folk remedy in Turkey. *Journal of Ethnopharmacology*.

[11] Saracoglu I, Inoue M, Calis I, Ogihara Y (1995). Studies on constituents with cytotoxic and cytostatic activity of two Turkish medicinal plants *Phlomis armeniaca* and *Scutellaria salviifolia*. *Biological and Pharmaceutical Bulletin*.

[12] Kyriakopoulou I, Magiatis P, Skaltsounis AL, Aligiannis N, Harvala C (2001). A new phenylethanoid glycoside with free-radical scavenging and antimicrobial activities from *Phlomis samia*. *Journal of Natural Products*.

[13] Marin PD, Veitch NC, Grayer RJ, Kite GC, Soković M, Janaćković P (2007). Flavonoids from *Phlomis fruticosa* (Lamiaceae) growing in Montenegro. *Biochemical Systematics and Ecology*.

[14] Adams RP (2007). *Identification of Essential Oil Components By Gas Chromatography-Mass Spectrometry*.

[15] Soković MD, Marin PD, Simić D, Knežević-Vukčević J, Vajs V, Petrović S (2002). Antimutagenic activity of essential oil and crude extract of *Phlomis fruticosa*. *Pharmaceutical Biology*.

[16] Başer KHC, Demirci B, Dadandi MY (2008). Comparative essential oil composition of the natural hybrid *Phlomis vuralii* dadandi (lamiaceae) and its parents. *Journal of Essential Oil Research*.

[17] Dapkevicius A, Venskutonis R, van Beek TA, Linssen PH (1998). Antioxidant activity of extracts obtained by different isolation procedures from some aromatic herbs grown in Lithuania. *Journal of the Science of Food and Agriculture*.

[18] Hatano T, Kagawa H, Yasuhara T, Okuda T (1988). Two new flavonoids and other constituents in licorice root: their relative astringency and radical scavenging effects. *Chemical and Pharmaceutical Bulletin*.

[19] Oyaizu M (1986). Studies on products of browning reactions: antioxidative activities of browning reaction prepared from glucosamine. *Japanese Journal of Nutrition*.

[20] Dinis TCP, Madeira VMC, Almeida LM (1994). Action of phenolic derivatives (acetaminophen, salicylate, and 5-aminosalicylate) as inhibitors of membrane lipid peroxidation and as peroxyl radical scavengers. *Archives of Biochemistry and Biophysics*.

[21] Slinkard K, Singleton VL (1977). Total phenol analyses: automation and comparison with manual methods. *American Journal of Enology and Viticulture*.

[22] Arvouet-Grand A, Vennat B, Pourrat A, Legret P (1994). Standardization of a propolis extract and identification of the main constituents. *Journal de Pharmacie de Belgique*.

[23] Basta A, Tzakou O, Couladis M (2006). The essential oil composition of *Phlomis cretica* C. Presl. *Flavour and Fragrance Journal*.

[24] Kirimer N, Başer KHC, Kürkcüoglu M (2006). Composition of the essential oil of *Phlomis nissolii* L. *Journal of Essential Oil Research*.

[25] Morteza-Semnani K, Moshiri K, Akbarzadeh M (2006). The essential oil composition of *Phlomis cancellata* Bunge. *Journal of Essential Oil Research*.

[26] Zhang Y, Wang ZZ (2008). Comparative analysis of essential oil components of three *Phlomis* species in Qinling Mountains of China. *Journal of Pharmaceutical and Biomedical Analysis*.

[27] Sarikurkcu C, Arisoy K, Tepe B, Cakir A, Abali G, Mete E (2009). Studies on the antioxidant activity of essential oil and different solvent extracts of *Vitex agnus castus* L. fruits from Turkey. *Food and Chemical Toxicology*.

[28] Gordon MH, Hudson BJF (1990). The mechanism of antioxidant action in vitro. *Antioxidants*.

[29] Halliwell B (1991). Reactive oxygen species in living systems: source, biochemistry, and role in human disease. *American Journal of Medicine*.

[30] Couladis M, Tzakou O, Verykokidou E, Harvala C (2003). Screening of some Greek aromatic plants for antioxidant activity. *Phytotherapy Research*.

